# Exception from Informed Consent in Emergency Care Research: Reports from a Workshop Addressing “Edge Cases”

**DOI:** 10.1002/eahr.70014

**Published:** 2025-12-31

**Authors:** Jeremy Brown, Neal W. Dickert, Robert Silbergleit

**Affiliations:** ^1^ Director of the Office of Emergency Care Research at the National Institutes of Health; ^2^ Professor in the Division of Cardiology in the Department of Medicine and the Emory Health Services Research Center at the Emory University School of Medicine; ^3^ Professor of emergency medicine at the University of Michigan Medical School

**Keywords:** human subjects research, research ethics, emergency setting, emergency care research, decisional incapacity, surrogate decision‐maker, legally authorized representative

## Abstract

The ethical challenges of enrolling critically ill patients into emergency care clinical trials without their consent remain, despite a 30‐year regulatory framework for conducting research in the emergency setting. In this series introduction, we outline some suggestions to improve studies conducted under the regulatory framework involving an exception from informed consent for emergency care research. These suggestions were made at a recent workshop sponsored by the National of Institutes of Health.

Respecting autonomy is a central component of respect for persons and drives the general recognition in human subjects research that “voluntary consent of the human subject is absolutely essential.”^1(p.1436)^ However, in medical emergencies, patients are often unconscious or otherwise incapable of making decisions about whether they want to participate in research to treat their condition, and surrogate decision‐makers are often not readily available to decide whether to enroll the patient in a study. Yet conducting research in the emergency care setting is essential to advance medical care for patients experiencing medical emergencies.

In 1996, the US Food and Drug Administration (FDA) issued regulations that permit, when certain conditions are met, researchers to enroll patients in emergency research without obtaining their informed consent to participate in the study.^2^ The regulations regarding “exception from informed consent” (EFIC) have intentionally strict requirements, largely because conducting research with people who did not provide consent to be a research participant should not be taken lightly, especially in serious medical situations. The EFIC regulations require, among other things, that (a) the patient's condition is life‐threatening and available treatments are unproven or unsatisfactory, (b) it is not feasible to obtain informed consent because the patient is incapacitated and the intervention must be delivered immediately, and (c) there is a prospect of direct benefit to the patient.

These regulations, and the companion guidance from the FDA,^3^ have provided a crucial framework to facilitate important emergency care research. But over the nearly thirty years since the EFIC regulations were implemented, it has become clear that there are ambiguities and challenges related to the regulations that require further ethical and legal clarification. In March 2024, the Office of Emergency Care Research at the National Institutes of Health (NIH) hosted a workshop, “Regulatory Determinations Related to Consent, EFIC, and Waiver of Consent in Emergency Clinical Trials,” to address some of these challenges. The workshop was attended by representatives of the FDA, the NIH, and institutional review boards; ethicists; and clinical researchers. Attendees participated in lively debates around several cases that were presented. Three papers in this journal, two in this issue^4^, ^5^ and the other forthcoming,^6^ capture important elements of that discussion, each highlighting an aspect of the EFIC regulations that is operationally or ethically challenging. The focus of the workshop was on “edge cases”: trials involving emergency situations in which research is needed to inform acute care but one or more elements of the EFIC framework do not clearly fit, seem out of place, or cause unanticipated problems. The workshop explored how regulations concerning informed consent, EFIC, and waivers or modifications of consent might be applied and interpreted in these difficult situations.

Importantly, the regulations and FDA guidance do not clarify the most appropriate way to approach patients who cannot fully engage in an informed consent process or surrogates who can be involved in decision‐making about enrollment. Indeed, in many (if not all) emergencies, capacity to fully engage in an informed consent process exists on a spectrum in which a threshold between a patient's incapacity and capacity to consent is indistinct or absent. With conditions like cardiogenic shock or stroke, patients on one end of the spectrum are either still able to provide meaningful informed consent or have a legally authorized representative (LAR) present who can do so immediately.

On the other end are patients who are obtunded or otherwise unable to communicate and who have no contactable LAR. Many other patients, however, lie somewhere between these extremes. They may not have the mental capacity (or the time) to give a fully informed consent, but perhaps they can give what we might call a partially informed consent. The FDA's current EFIC guidance^3(p.14)^ suggests that, at a minimum, patients be offered an opportunity to object to enrolling in emergency care research, but little is known about how to operationalize this approach. There are also related instances, such as in acute pain research, where regulatory requirements regarding the practicability of obtaining informed consent (and the potential to enroll patients across a spectrum of capacity to consent) have inadvertently compelled researchers to modify protocols in ways that are ill‐suited to the context and scientifically or ethically problematic, such as restricting enrollment to only the most severely ill patients.

Other edge cases discussed were related to risk assessment in the context of trials comparing existing therapies. The EFIC regulations were written with studies that test novel therapies primarily in mind. However, risk assessment in the context of comparative effectiveness research (CER) can be very different. Consider the care of patients with acute delirium, a group for whom informed consent in an emergency setting seems implausible. If a trial involves assignment to one among several standard treatment options and the patient could be expected to receive the very same medications

**The set of papers in this journal reporting about the issues and challenges the workshop addressed start to better define, and perhaps carefully and cautiously expand, the ethical and practical contours of the edges around the EFIC regulations.**

even if they had not been part of a clinical trial, it is not clear what specific risks the trial itself could present. Increasingly, there has been a move to consider CER trials in many health care contexts to present no more than minimal risk, and a recent FDA final rule^7^ explicitly recognizes this designation, which also may permit a waiver or alteration of consent. However, it is unclear how traditional concepts of minimal risk map onto emergency care settings in which background risks of major morbidity and mortality are high and in which interventions are being evaluated for their ability to reduce these important outcomes.

A final set of challenges representing a form of an edge case has to do with representation. Minority or disadvantaged populations are more likely to be enrolled in some EFIC trials. For example, trauma is often more prevalent in socioeconomic areas where racial and ethnic minority populations are found in higher concentrations. Enrolling trauma patients under EFIC would then result in the enrollment of a far higher number of patients from these populations than patients who are members of a racial or ethnic minority. The workshop stimulated the discussion of clinical trial scenarios that do not neatly fall into current EFIC regulations and, we believe, represented a critical step forward. We have nearly 30 years of experience with the conduct of EFIC trials, especially in populations such as patients with cardiac arrest and acute traumatic brain injury. The set of papers in this journal reporting about the issues and challenges the workshop addressed start to better define, and perhaps carefully and cautiously expand, the ethical and practical contours of the edges around the EFIC regulations. The workshop was a call to continue to address nuanced issues that are critical to advancing the ethical conduct of research in many acute and emergency conditions.
